# Conscientiousness and Smoking: Do Cultural Context and Gender Matter?

**DOI:** 10.3389/fpsyg.2020.01593

**Published:** 2020-07-07

**Authors:** Chioun Lee, Manjing Gao, Carol D. Ryff

**Affiliations:** ^1^Department of Sociology, University of California, Riverside, Riverside, CA, United States; ^2^Institute on Aging and Department of Psychology, University of Wisconsin-Madison, Madison, WI, United States

**Keywords:** culture, health, smoking, stigma, gender, Japan, United States

## Abstract

Prior studies have found that conscientiousness has a protective effect against smoking, but evidence for this relationship mostly comes from Western contexts. In societies where smoking is pervasive and less stigmatized, the protective effect of conscientiousness on smoking may be less evident. Moreover, whether smoking is viewed as normal or deviant also may vary by gender norms attached to smoking. Using surveys of Midlife Development in the United States (MIDUS) and Japan (MIDJA), we examined patterns in the association between conscientiousness and smoking status (never, former, current) for men and women. We found that in the United States, where the social unacceptability of smoking has dramatically increased, there is an inverse association between conscientiousness and smoking status for both genders. In Japan, where the stigma attached to smoking operates for women but not men, the association between conscientiousness and smoking status varies by gender. For Japanese men, levels of conscientiousness do not differ across smoking statuses. For Japanese women, those who formerly smoked show lower levels of conscientiousness than those who never smoked and those who currently smoke. We interpret these findings in light of differing cultural and historical backgrounds of smoking for men and women.

## Introduction

Personality traits are well-established predictors of health and health behaviors. Cumulative evidence shows that the Big Five personality traits—extraversion, neuroticism, agreeableness, conscientiousness, and openness to experience—are linked to central health-related behaviors (Hakulinen and Jokela, 2018). Among the Big Five, conscientiousness is a trait related to individual differences in the likelihood of following socially prescribed norms and rules (John and Srivastava, 1999). There is fairly consistent evidence that individuals who have higher levels of conscientiousness are likely to abstain from the leading behavioral contributors of mortality, such as smoking (Kubička et al., 2001; Bogg and Roberts, 2004; Hakulinen et al., 2015). Yet, the evidence for this association mostly comes from Western societies that emphasize individual choice and where smoking is more stigmatized (Ando et al., 2007; Gelfand et al., 2011). Little is known about societies that attach more importance to group/societal values and where smoking is more pervasive and less stigmatized. Further, while the deviant nature of smoking differs by gender in some societies (Amos, 2000; Greaves, 2015), it remains unclear how gender may moderate the link between conscientiousness and smoking.

Using data from surveys of Midlife Development in the United States (MIDUS) and Japan (MIDJA), we investigate whether the association between conscientiousness and smoking varies by culture and gender. The United States and Japan provide interesting contexts because both countries have similar levels of industrialization, but differ substantially in the political and sociocultural structures that may facilitate or inhibit smoking behaviors of men versus women. Our study is among the first to assess the role of sociocultural context in moderating the association between conscientiousness and smoking. The findings from this study provide insight into how the effect of personality traits on health-related behaviors varies by culture and gender.

## Background

### Conscientiousness and Smoking

Individual differences in personality traits predict various health outcomes (Christensen et al., 2002; Ploubidis and Grundy, 2009; Strickhouser et al., 2017). In particular, conscientiousness—the trait of being goal-directed, of following social norms, and of greater impulse control, cautiousness, self-discipline, and desire for orderliness (John and Srivastava, 1999; Roberts et al., 2009) has a particularly protective effect against smoking, the leading cause of preventable death globally (World Health Organization [WHO], 2017). In a meta-analysis of nine cohort studies from Australia, Germany, the United Kingdom, and the United States, Hakulinen et al. (2015) found that both former and current smokers had lower levels of conscientiousness than never-smokers. A longitudinal study from the Czech Republic further indicated that individuals with higher levels of conscientiousness in childhood were less likely to be regular smokers in young adulthood (Kubička et al., 2001). Other work focusing on midlife adults in the United States has shown that conscientiousness has protective effects against smoking initiation and persistence over a 10-year period (Turiano et al., 2012; Zvolensky et al., 2015). Overall, prior work indicates that individuals with higher levels of conscientiousness are less likely to engage in health-risk behaviors, such as smoking.

However, the generalizability of the conscientiousness–health link is an open question because evidence for the association comes mostly from Western, individualistic societies, which are known to emphasize the importance of personal choice (Ando et al., 2007; Gelfand et al., 2011). Although evidence exists, there are relatively few studies from collectivistic societies (Yoshimura, 2000; Leung et al., 2013; Abe et al., 2019), which attach more importance to groups and societal values (Gelfand et al., 2011). The association between conscientiousness and risky health behaviors might be attenuated if the behavior itself is considered less socially deviant (Bogg and Roberts, 2004). For example, the probability of smoking increases in places where many people smoke because it makes smoking seem more acceptable (Harbour, 2012; Corsi et al., 2013). Indeed, researchers have found that smokers living in neighborhoods where smoking is prevalent perceive lower levels of stigma compared to those living in neighborhoods where smoking is relatively rare (Stuber et al., 2008).

In addition to playing a role in defining smoking as a normative versus deviant behavior, culture may be linked to gendered patterns of smoking (Amos, 2000; Greaves, 2015). In contrast to “sex,” which refers to biological aspects of maleness and femaleness, “gender” refers to socially constructed and enacted roles and behaviors which arise in a historical and cultural context and differ across societies and throughout time (National Institute of Health Office of Research on Women’s Health, 2020). Smoking is considered a masculine behavior only appropriate for men in some cultures (Hirokawa et al., 2006). Indeed, during the late Victorian periods in Britain, smoking among women was stigmatized and confined to those “on the fringe of respectable society” (Hilton, 1995). However, current research on the association between conscientiousness and smoking has seldom considered the role of gender. It thus remains unclear how gender may moderate the link between conscientiousness and smoking (Kashdan et al., 2005; Hampson et al., 2006). We further consider the role of cultural context, given that norms about smoking may differ by gender in some cultures but not others, and such norms may change over time.

### Societal and Historical Contexts of Smoking

Tobacco consumption is one of the leading preventable causes of mortality globally, accounting for more than seven million deaths each year (World Health Organization [WHO], 2017). The prevalence of smoking has fallen in both the United States and Japan over the past three decades, and recent data show that around one fifth of the population in both counties has ever smoked (World Bank, 2016). However, the prevalence of daily smokers and annual cigarette consumption per capita remains substantially higher in Japan compared to the United States; for example, 18.2% versus 11.4% in 2015 (OECD, 2020) and 1,904 versus 1,235 in 2010 (Hoffman et al., 2019), respectively. Differing historical and political circumstances have shaped these differences in levels of smoking consumption.

In the United States, the 1964 Surgeon General’s report on Smoking and Health marked a watershed in shifting public attitudes about smoking from a “natural accompaniment of work and play” to a health risk behavior (U.S. Department of Health Human Services, 1989; Cummings, 1997). Widespread publicity about findings in the Surgeon General’s report fueled a major political agenda through which numerous policies were established to address the hazards of smoking (Rabin and Sugarman, 1993; Cummings, 1997). In the 1990s, smoking was widely perceived as unhealthy and was becoming increasingly unacceptable. Anti-smoking policies and movements continued to drive down smoking rates through the 2010s (Cummings and Proctor, 2014). In contrast, tobacco control policies in Japan largely failed because of political alliances between the tobacco industry and the government (Levin, 2013). For example, from 1883 to 1985, the Japanese government held a monopoly on the manufacture and sale of tobacco products (Honjo, 2000). Although the tobacco industry has since become privatized, the government of Japan is still indirectly involved, owning about one third of common stock in tobacco companies in 2004 (Levin, 2013).

Moreover, foreign interests have helped facilitate the prevalence of smoking in Japan. In the United States, after the 1964 Surgeon General’s Report and the ensuing growth of anti-smoking movements, demand for tobacco products decreased (Honjo, 2000). Seeking new markets, United States tobacco companies and the United States government pressured Japan to ease restrictions on United States tobacco imports (Honjo, 2000). The Japanese government adopted several measures during the 1980s to reduce cigarette prices, such as abolishing the tariff, expanding sales networks, and abolishing restrictions on the advertising and promotion of tobacco (Honjo, 2000). Such political arrangements may have inhibited policy interventions, such as bans on smoking in indoor public places and nationwide mandatory clean air laws. Stated otherwise, the government and tobacco industry in Japan jointly fostered a smoke-friendly environment.

At the same time, Japan tends to have stronger norms and a lower tolerance of deviant behavior than the United States (Gelfand et al., 2011). Conformity to sameness within intragroup relations is valued in Japan, whereas individual rights are more valued in the United States (Ando et al., 2007; Morrow and Barraclough, 2010). Thus, compared to the United States, the collective-oriented culture of Japan may confine individuals to a more limited range of appropriate behaviors, leaving less room for individual discretion (Gelfand et al., 2011). Japanese culture may also place a heavier burden on individuals to follow social norms, providing more social incentives as well as social pressures for individuals to smoke. As a result, tobacco use has been viewed as more of an issue of polite manners than a health problem (Kanamori and Malone, 2011). That is, many Japanese consider it a matter of social etiquette to ask the permission of others nearby to smoke, and it is believed to be polite to grant permission (Yorozu and Zhou, 2002). Given that smoking can also be a way of starting connections with others (Morrow and Barraclough, 2010), many young and middle-aged Japanese adults might be “social smokers,” that is, those who smoke as a gesture to maintain social relationships and follow social norms. Under such circumstances, even those who are conscious about the health hazards of smoking may nonetheless accept an offer to smoke.

### Bringing Emphasis to Gender and Smoking

In 2005, around one fifth of the population (ages 15+) in both counties had ever smoked, but what makes these comparable levels of smoking interesting is the wider gender gap in Japan. For example, in 2015, 25.1% of men and 19.6% of women smoked in the United States, compared to 34.7% of men and 11.4% of women in Japan (World Bank, 2016). The markedly higher smoking prevalence among men in Japan reflects and/or reinforces the conception that smoking is normal among men, but not among women. For generations, smoking has been associated with masculinity (Hirokawa et al., 2006), while women’s smoking has been seen as a deviant behavior (Gilman and Xun, 2004). For example, it was common to associate women-smokers with prostitutes in the early 19th century (Nagashima, 2007). In the past, young women-smokers were regarded as showy and slovenly by both women and men (Tanikawa, 1995).

As women’s suffrage proceeded throughout the mid-20th century, the stigma associated with their smoking decreased (Marugame et al., 2006), and smoking came to be viewed as a symbol of their emancipation and empowerment (Fukuda et al., 2005; Hitchman and Fong, 2011). However, as awareness of the health effects of smoking grew, it also was increasingly seen as a risky health behavior for women and their offspring (Nishi et al., 2005). Moreover, with their growing social positions and power, Japanese women have increased their influence on social workplace smoking by fighting for smoke-free environments (Yoshimura, 2000). Therefore, the stigma attached to women’s smoking in Japan may continue.

### Aims and Hypotheses of the Present Study

Using samples from the United States and Japan, we investigate how associations between conscientiousness and smoking vary by sociocultural context and gender. Based on the above historical and cultural perspectives on smoking, we expect that in the United States, there will be no gender difference regarding the association between conscientiousness and smoking. For both men and women, current smokers will show less conscientiousness than former smokers, with non-smokers showing the highest levels of conscientiousness (Hypothesis 1a). Given that individuals with lower levels of conscientiousness may have more problems with impulse control and are less likely to succeed in quitting smoking (Leung et al., 2013; Zvolensky et al., 2015), we expect that former smokers will have higher levels of conscientiousness than current smokers. In contrast, we hypothesize that in Japan where smoking is more prevalent (particularly for men), less stigmatized, and considered an everyday social activity, the protective effect of conscientiousness on smoking will be less evident. Given the gendered cultural contexts of smoking in Japan, among Japanese women, current or former smokers will be less conscientious than never-smokers (Hypothesis 2a); among men, current or former smokers will be more conscientious than never-smokers (Hypothesis 2b). The United States and Japan provide interesting contexts to investigate these hypotheses because both countries have similar levels of industrialization but differ substantially in the political and sociocultural structures that may facilitate or inhibit micro-level factors (personality traits) related to smoking among men and among women.

## Materials and Methods

### Sample

We compare data from the Surveys of MIDUS and MIDJA. MIDUS is a national survey that investigates the role of social, psychological, and behavioral factors in explaining variation in physical and mental health among United States residents. English-speaking, non-institutionalized adults aged 25–74 in the 48 states were first interviewed in 1995–1996 (*n* = 7,108) and were followed up in 2004–2006 (*n* = 4,936). The MIDUS series also includes a sister survey conducted in Japan, Midlife in Japan (MIDJA), to investigate cultural differences in the effects of psychosocial factors on health. The MIDJA study is based on a random sample of Japanese participants (*n* = 1,027 aged 30–79) from the Tokyo metropolitan area and was conducted first in 2008, with a follow-up in 2012. Although MIDJA was not nationally representative, it provides important insights for the context of Japan. To pursue better historical comparability of the United States participants with their Japanese counterparts, we focus on the Wave 2 sample of MIDUS (2004–2006) and Wave 1 of MIDJA. Our analytic sample includes 1,027 Japanese adults who participated in MIDJA Wave 1 and 4,041 American adults who completed a self-report questionnaire in MIDUS Wave 2.

### Measures

Both surveys contain personality traits, status of cigarette smoking, and sociodemographic characteristics. Assessment of personality traits in the Midlife Development Inventory Personality Scales (MIDI) was based on the Big Five factor model (John and Srivastava, 1999). *C****onscientiousness*** is an index score generated by averaging five items [organized, responsible, hardworking, thorough, and careless (reverse coded)]. Respondents were asked how much each of the four items describes them on a scale ranging from 1 (not at all) to 4 (a lot). Higher scores on the index indicate higher levels of conscientiousness. Cronbach alpha reliabilities (α) were 0.66 for MIDJA and 0.69 for MIDUS. The average score of conscientiousness differed by society, with a higher score for the United States (mean = 3.38) than Japan (mean = 2.60), which is consistent with prior studies that reported that Japanese/Asian participants provide moderate rather than strong (i.e., more idealized) self-report ratings than United States participants (Schultz and Schultz, 2017). We standardized the index of conscientiousness to have a mean of zero and standard deviation of one in each sample to facilitate cross-cultural comparisons.

Given that other personality traits are also associated with smoking (Hakulinen et al., 2015) we included ***neuroticism*** [worrying, nervous, irritable, calm (reverse coded); α = 0.74 for MIDUS and α = 0.51 for MIDJA], ***extraversion*** (outgoing, friendly, lively, active, talkative; α = 0.76 for MIDUS and α = 0.83 for MIDJA), ***openness*** (creative, imaginative, intelligent, curious, broad-minded, sophisticated, adventurous; α = 0.77 for MIDUS and α = 0.84 for MIDJA), and ***agreeableness*** (helpful, warm, caring, softhearted, sympathetic; α = 0.80 for MIDUS and α = 0.87 for MIDJA).

#### Cigarette Smoking

Participants from both MIDUS and MIDJA were asked a series of questions regarding their smoking habits: (1) whether they had ever smoked cigarettes, (2) whether they had ever smoked regularly, and (3) whether they currently smoke cigarettes regularly. Based on this information, we created a set of three dummy variables for smoking statuses: *never smokers* (never smoked regularly or never smoked at all), *former smokers* (smoked regularly in the past, but not at the time of the survey), and *current smokers*.

In addition, we controlled for age, marital status, and education for both MIDUS and MIDJA samples and for race for the MIDUS sample only. ***Age*** was included as a continuous variable. ***Marital status*** was measured by respondents’ current marital status at the time, recoded to 1 (married) and 0 (other statuses). For ***race***, respondents were asked to indicate the best description of their racial category. Responses were recoded as 1 (white) and 0 (non-white). ***Educational level*** was determined on the basis of the highest achieved grade for both samples (1 = less than high school, 2 = high school, 3 = some college, and 4 = BA or higher).

### Analytic Strategies

**FIGURE 1:**
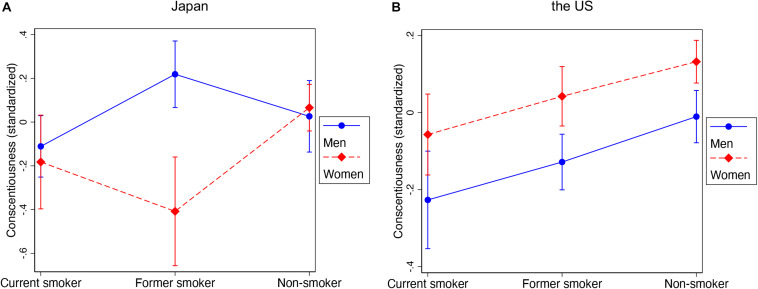
Conscientiousness across smoking statuses, by culture and gender. The figure is based on the results of ANOVA in each sample with a gender interaction term. **(A)** Gender Difference in the Levels of Conscientiousness across Smoking Status in Japan. **(B)** Gender Difference in the Levels of Conscientiousness across Smoking Status in United States.

To test our hypotheses, we used one-way ANOVA to determine whether there were significant differences in the means of conscientiousness across smoking groups (never, former, current smokers) and whether there were gender differences in the association. When F-tests in ANOVA were statistically significant, we performed *post hoc* pairwise comparisons with the Tukey–Kramer test for unequal sample size. Correcting for multiple comparisons reduces the possibility of a significant finding being attributed to mere chance (Tukey, 1949). We analyzed each sample (United States versus Japan) separately and used margin commands in STATA to illustrate the results (Figure 1). Then, we used multinomial logistic regression to estimate the association between conscientiousness and smoking status by using never smoked as a baseline category of the outcome variable versus former smoking and current smoking (Table 2). Each sample was analyzed separately with gender-stratified models, and we tested for gender differences by pooling data from both genders and testing gender interaction terms. Finally, we performed sensitivity analysis to show that our results are robust to potential measurement issues regarding conscientiousness related to health behaviors (Appendix Table 1). All multivariate models control for the other four personality traits and sociodemographic characteristics.

Approximately 7% of MIDUS and 5% of MIDJA respondents had missing data for at least one variable of interest. Using Stata’s *ice* command (Royston and White, 2011) we implemented 50 imputations to predict missing variables by generating imputed values. All analyses were implemented using Stata 15.0 (StataCorp, 2017).

## Results

Descriptive statistics [mean (SD) or proportion] for all variables are presented by sample and gender in Table 1. The smoking prevalence among Japanese men was higher than that among United States men. Only 28% of Japanese men in the sample had never smoked, compared to 46% of United States men. Further, there is a clear gendered pattern of smoking in Japan, with the prevalence of non-smokers being much higher among women (69%) than men (28%). In contrast, the gender gap in smoking statuses was much smaller in the United States, with 55% of women never smoking, compared to 46% of men.

**TABLE 1:** Means (and standard deviations) or proportions for all variables used in analysis by culture and gender, MIDUS (2004–2006) and MIDJA (2008).

	MIDJA (Japan)		MIDUS (the United States)	
	Men (*n* = 505)	Women (*n* = 522)	Men (*n* = 1,802)	Women (*n* = 2,239)
**Smoking status**				
Never smoked	0.28	0.69	0.46	0.55
Former smokers	0.33	0.14	0.41	0.29
Current smokers	0.39	0.17	0.13	0.16
**Personality traits**				
Conscientiousness	2.62 (0.57)	2.59 (0.52)	3.35 (0.46)	3.42 (0.46)
Openness	2.26 (0.62)	2.11 (0.59)	2.93 (0.52)	2.88 (0.55)
Neuroticism	2.16 (0.55)	2.06 (0.55)	1.99 (0.61)	2.13 (0.64)
Extraversion	2.39 (0.67)	2.46 (0.69)	3.06 (0.57)	3.15 (0.57)
Agreeableness	2.63 (0.63)	2.64 (0.63)	3.29 (0.52)	3.58 (0.44)
**Sociodemographic characteristics**				
Age	54.69 (14.41)	54.04 (13.86)	56.53 (12.22)	56.00 (12.51)
Race/ethnicity white = 1)	–	–	0.95	0.93
Marital status (currently married = 1)	0.73	0.66	0.79	0.65
** Education**				
Less than high school	0.14	0.12	0.06	0.06
High school	0.25	0.35	0.23	0.30
Some college	0.15	0.35	0.26	0.31
BA or higher	0.46	0.18	0.45	0.33

Figure 1 shows the results of ANOVA to investigate the differences in conscientiousness among current smokers, former smokers, and those who never smoked in the United States and Japan. For both samples, levels of conscientiousness significantly varied by smoking status. In the United States sample, those who never regularly smoked had the highest levels of conscientiousness, former smokers had the second highest levels of conscientiousness, and current smokers had the lowest levels of conscientiousness, regardless of gender. After performing multiple comparisons, we found that levels of conscientiousness are statistically different between non-smokers and current smokers for both genders.

In contrast, the Japanese sample followed a different pattern and varied by gender: for men, former smokers had *higher* levels of conscientiousness (0.22 SD above the mean) than never smokers (close to the mean) and current smokers (0.11 SD below the mean), although the difference is statistically significant only between former smokers and current smokers (*p* < 0.05). For women, former smokers had the *lowest* level of conscientiousness (0.41 SD below the mean), compared to current smokers (0.18 SD below the mean) and those who never smoked (close to the mean). The mean difference between former smokers and never-smokers was statistically significant (*p* < 0.05). We also found a significant gendered pattern in terms of the mean difference between current smokers and former smokers (*p* < 0.01). For women, former smokers had levels of conscientiousness that were 0.22 SD lower than current smokers, while for men, former smokers had levels of conscientiousness that were 0.33 SD higher than current smokers.

Table 2 presents the results from the multinomial logistic regression used to investigate our hypotheses. It shows that in the United States, lower levels of conscientiousness predict increased odds of being in the current smoker group compared to the never-smoker group for both men and women, even after controlling for the other personality traits, education, age, race, and marital status. For men, a one SD increase in conscientiousness is associated with a 0.24 decrease in the relative log odds of being current smokers versus never-smokers (*p* < 0.01) and a 0.12 decrease in the relative log odds of being former smokers versus never-smokers (*p* < 0.05). For women, higher levels of conscientiousness decrease the risk of becoming former smokers and current smokers. For former smokers, a one SD increase in the level of conscientiousness is associated with a 0.11 decrease in the relative log odds of being former smokers versus non-smokers (*p* < 0.05). Similarly, a one SD increase in conscientiousness is associated with a 0.12 decrease in the relative log odds of being current smokers versus non-smokers (*p* < 0.1).

**TABLE 2:** Summary of multinomial logistic regression predicting smoking status, by culture and gender.^a^

	Japang				The United States			
	Model 1a: Men (*n* = 505)		Model 1b: Women (*n* = 522)		Model 2a: Men (*n* = 1,802)		Model 2b: Women (*n* = 2,239)	
	Former smokers B (SE)	Current smokers B (SE)	Former smokers B (SE)	Current smokers B (SE)	Former smokers B (SE)	Current smokers B (SE)	Former smokers B (SE)	Current smokers B (SE)
**Personality^b^**								
Conscientiousness	0.07 (0.15)	−0.16(0.15)	−0.44(0.18)*	−0.20(0.17)	−0.12(0.06)*	−0.24(0.08)**	−0.11(0.06)*	−0.12(0.07)^#^
Openness	−0.26(0.19)	−0.27(0.18)	−0.07(0.21)	0.13 (0.19)	0.21(0.07)**	0.31(0.10)**	0.23(0.06)***	0.20(0.08)*
Neuroticism	0.32(0.13)*	0.29(0.13)*	0.22 (0.15)	0.24(0.14)^#^	0.14(0.06)*	−0.004(0.08)	0.14(0.05)**	0.27(0.07)***
Extraversion	0.33(0.19)^#^	0.63(0.18)***	64(0.23)**	0.54(0.19)**	−0.15(0.07)*	−0.11(0.10)	−0.02(0.06)	−0.11(0.08)
Agreeableness	0.12 (0.20)	0.02 (0.20)	−0.19(0.22)	−0.38(0.20)^#^	0.09 (0.06)	0.02 (0.09)	0.01 (0.07)	0.16(0.09)^#^
**Education^c^**								
High school	−0.40(0.43)	0.10 (0.42)	0.28 (0.60)	−0.29(0.45)	−0.36(0.28)	−0.36(0.34)	−0.03(0.23)	−0.63(0.24)*
Some college	−0.25(0.50)	−0.49(0.48)	0.33 (0.62)	−0.42(0.47)	−0.60(0.27)*	−0.91(0.34)**	−0.05(0.23)	−0.78(0.25)**
BA or higher	−0.55(0.40)	−0.64(0.41)	−0.38(0.69)	−10.36(0.55)*	−1.33(0.27)***	−2.15(0.34)***	−0.42(0.24)^#^	−2.09(0.28)***
Age	0.02(0.01)*	−0.02(0.01)*	−0.02(0.01)	−0.04(0.01)***	0.05(0.005)***	−0.02(0.01)*	0.01(0.004)	−0.04(0.01)***
White	− −	− −	− −	− −	0.35 (0.26)	0.22 (0.35)	0.48(0.21)*	0.54(0.26)*
Married	0.91(0.30)**	0.42 (0.26)	−0.16(0.30)	−0.38(0.27)	0.02 (0.14)	−0.38(0.18)*	−0.16(0.11)	−0.88(0.13)***

^a^The models investigate whether those who used to smoke regularly and those who currently smoke regularly differ from those who never smoked regularly (reference group). ^b^All personality traits are standardized at mean (SD = 1.0). ^c^Middle school or lower is the reference group for education. ^#^p < 0.1, *p < 0.05, **p < 0.01, ***p < 0.001.

In contrast, in Japan, we found that conscientiousness is only significantly associated with smoking status for women after controlling for all covariates. For Japanese women, a one SD increase in conscientiousness predicts a 0.44 decrease in the relative log odds of being a former smoker compared to never having smoked (*p* < 0.05). However, there is no significant association between conscientiousness and smoking status for Japanese men.

### Sensitivity Analysis

The results in Table 2 may be subject to issues regarding measurement validity of conscientiousness across different cultures and societies. That is, our findings would be invalidated if the index of conscientiousness is related to healthy behaviors in one society (the United States) but not in the other society (Japan). To address this concern, we investigated individuals’ participation in routine annual physical exams, which are considered a preventive health behavior globally, regardless of gender. The associations between conscientiousness and a regular check-up are presented in Appendix Table 1. Unlike the smoking outcomes, we found a similar pattern for both men and women in both societies. That is, individuals who had higher levels of conscientiousness, regardless of gender and society, were more likely to participate in regular physical check-ups even after controlling for the other personality traits, education, age, marital status, and race. Thus, individuals with higher levels of conscientiousness tend to adopt health-promoting behaviors. In contrast, the meaning of smoking largely depends on the sociocultural context. Thus, the sensitivity analyses show that our findings on smoking are robust to concerns regarding measurement validity.

## Discussion

This study advances previous research that has examined the link between conscientiousness and smoking in several ways. First, the majority of previous studies have focused on Western samples (Turiano et al., 2012; Zvolensky et al., 2015), thus leaving unanswered the question of whether conscientiousness affects smoking differently across various sociocultural contexts. Our study adopted the definition of conscientiousness as a personality trait characterized by following socially prescribed norms (Roberts et al., 2009). Drawing on societal and historical perspectives, we proffered that social acceptance of smoking varies by context (Echeverría et al., 2015). In particular, our study explored how sociocultural conditions may interact with personality traits to shape health behaviors by comparing Japan—a collectivistic society that attaches more importance to groups and societal values and has a high prevalence of smoking particularly for men—and the United States—the exemplar individualistic society in which the social unacceptability of smoking has dramatically increased. Second, few previous studies have investigated whether and how gender may moderate the association between personality and smoking (e.g., Friedman et al., 1993; Kashdan et al., 2005; Hampson et al., 2006). Our study gives prominence to gendered social norms in Japan regarding smoking behaviors.

Our analyses yielded several interesting findings. First, we found that the association between conscientiousness and smoking differs by society and gender. As expected, lower levels of conscientiousness are associated with increased odds of being in the current smoker group compared to the never-smoker group for both men and women in the United States. Changes in attitudes and policies regarding smoking in the United States (e.g., smoke-free air laws and the social unacceptability of smoking) have occurred in tandem with an overall decrease in the number of smokers (Warner, 2014). As such, smoking has been denormalized and is less considered as a “social activity” (Burns, 2014). United States public health officials have implemented comprehensive tobacco control programs, such as restrictions on smoking in worksites and public places, tobacco taxation, and mass-media campaigns (Centers for Disease Control and Prevention, 2017). In this social and cultural environment, the personality trait of conscientiousness is aligned with changing societal attitudes about smoking. That is, both men and women who have higher levels of conscientiousness have more negative attitudes toward smoking and are less likely to smoke.

In contrast, there is no significant association between conscientiousness and smoking status for Japanese men. The collective-oriented culture in Japan may put more emphasis on following social norms and leave less room for individual discretion. Although the Japanese public has become more wary of smoking over the past decade (Goto et al., 2011), anti-smoking policies (e.g., restrictions on advertisements for tobacco products and smoking bans in public places) and cultural disapproval of smoking are more lax in Japan than in the United States (Fukuda et al., 2005), contributing to the relatively higher prevalence of smoking, particularly for men. Many young and middle-aged men in Japan may be “social smokers”—i.e., may smoke in social contexts to gain peer acceptance and social connections.

Further, our results suggest that gender plays different roles in patterns of smoking across societies. For men, smoking is less disapproved and more normalized in Japan than in the United States. As such, Japanese men who follow societal values are likely to choose to smoke socially rather than abstain. In contrast, women’s smoking is more stigmatized in Japan than the United States, particularly among older generations. Although the social stigma attached to women smokers may have relaxed recently as the socioeconomic status of women in Japan has increased (Fukuda et al., 2005), women who follow social norms are still more likely to be non-smokers. As such, we found that among Japanese women, former smokers have lower levels of conscientiousness than current and never smokers. To the extent that smoking is seen as a masculine behavior at odds with the expression of femininity (Gilman and Xun, 2004; Hirokawa et al., 2006), women who have high levels of conscientiousness may be more likely to distance themselves from smoking, a behavior predominantly for men in Japan.

### Limitations and Conclusions

Several limitations of our study should be noted. By employing a cross-sectional design, we have assumed that culture and personality traits are “fixed” (not changing or dynamic), although it is known that norms regarding smoking have changed over time. For some, the norms in place when smoking commenced may more strongly influence their smoking behaviors than norms at the time of MIDUS and MIDJA surveys in the mid or late 2000s. That is, our analyses cannot address how changing societal contexts relate to norms across time. Moreover, research indicates that personality traits vary by both ethnic groups and cultures related to geographical locations (Allik and Mccrae, 2004; Brown et al., 2005). Given that the majority of the MIDUS sample consists of European Americans while the majority of MIDJA consists of Asians, our findings across cultures might partially reflect ethnic differences. Third, our study lacks detailed and complex measures of smoking. For example, we suggested that many Japanese men may tend to be social smokers, but we have no data on whether they restrict their tobacco use to social situations. We also lack the data to measure abstinence duration for former smokers. Future research may use such an indicator to better capture how personality traits related to impulse control (i.e., conscientiousness) are linked to more nuanced smoking status.

Third, our findings are subject to omitted variable bias. For example, there might be other micro-level factors (genetics) in addition to personality traits that affect smoking behaviors (Loukola et al., 2014). Moreover, there was no measurement of nicotine dependence in MIDUS and MIDJA. Prior studies reported that personality traits (conscientiousness and neuroticism) are significantly related to nicotine dependence (Choi et al., 2017) and that the likelihood of quitting smoking is inversely related to the severity of nicotine dependence (Kozlowski et al., 1994; Siqueira et al., 2001; Baker et al., 2007), suggesting that nicotine dependence may play a role in the association between personality traits and smoking. Future studies would benefit from including more extensive measures of covariates. Finally, although we recognize that there are cohort differences in the prevalence of smoking, we lack the statistical power to analyze such differences; the sample size of smokers, particularly among Japanese women, is too small. Thus, longitudinal research is needed to better reveal the dynamics between macro-level sociocultural factors and micro-level personality traits.

Despite these limitations, our study is the first to document the influence of cultural context and gender on the association between the personality trait of conscientiousness and the health behavior of smoking. That is, the association between conscientiousness and smoking may be suppressed or facilitated by macro-level sociocultural factors. Our findings thus situate individuals in their social contexts, thereby emphasizing the “embeddedness” of social actors. By providing insight into different gendered patterns of smoking in different societies, our study calls for smoking interventions that are gender-sensitive.

## Data Availability Statement

The datasets generated for this study are available on request to the corresponding author.

## Ethics Statement

MIDUS data collection is reviewed and approved by the Education and Social/Behavioral Sciences and the Health Sciences IRBs at the University of Wisconsin-Madison. Written informed consent to participation in this study was provided by all participants of MIDUS.

## Author Contributions

CL initiated and designed the study, conducted the analysis, and wrote the manuscript. MG conducted the analysis and drafted the theoretical foundation of the manuscript under the supervision of CL. CR helped to formulate the research questions and provided critical feedback on the entire manuscript. All authors contributed to the article and approved the submitted version.

## Conflict of Interest

The authors declare that the research was conducted in the absence of any commercial or financial relationships that could be construed as a potential conflict of interest.

## Appendix

**APPENDIX TABLE 1:** Summary of logistic regression predicting participation in routine physical exam, by culture and gender.

	Japan		The United States	
	Model 1a: Men (*n* = 505)	Model 1b: Women (*n* = 522)	Model 2a: Men (*n* = 1,802)	Model 2b: Women (*n* = 2,239)

	B (SE)	B (SE)	B (SE)	B (SE)
**Personality^a^**				
Conscientiousness	0.09 (.13)	0.30(0.12)*	0.24(0.06)***	0.23(0.07)***
Openness	−0.28(0.15)^#^	−0.04(0.14)	−0.20(0.08)**	−0.13(0.08)^#^
Neuroticism	−0.03(0.10)	0.01 (0.10)	0.03 (0.06)	0.15(0.07)*
Extraversion	0.29(0.15)^#^	0.11 (0.14)	0.12 (0.07)	0.15(0.08)^#^
Agreeableness	0.10 (0.17)	−0.20(0.15)	0.08 (0.07)	0.08 (0.08)
**Education^b^**				
High school	0.41 (0.32)	0.26 (0.31)	0.54(0.27)*	0.32 (0.25)
Some college	0.20 (0.37)	0.74(0.34)*	0.66(0.26)*	0.67(0.26)**
BA or higher	1.08(0.32)***	1.26(0.40)**	0.86(.26)**	1.03(0.27)***
Age	0.01 (0.01)	0.01 (0.01)	0.07(0.01)***	0.02(0.01)***
White	− −	− −	−0.07(0.26)	0.004 (0.25)
Married	−0.04(0.23)	−0.15(0.20)	0.21 (0.14)	0.19 (0.14)

*^a^Middle school or lower is the reference group for education. ^b^All personality traits are standardized at mean (SD = 1.0). ^#^p < 0.1, *p < 0.05, **p < 0.01, ***p < 0.001.*
